# Little Evidence of Subclinical Avian Influenza Virus Infections among Rural Villagers in Cambodia

**DOI:** 10.1371/journal.pone.0097097

**Published:** 2014-05-12

**Authors:** Gregory C. Gray, Whitney S. Krueger, Channimol Chum, Shannon D. Putnam, Thomas F. Wierzba, Gary L. Heil, Benjamin D. Anderson, Chadwick Y. Yasuda, Maya Williams, Matthew R. Kasper, Vonthanak Saphonn, Patrick J. Blair

**Affiliations:** 1 College of Public Health and Health Professions and Emerging Pathogens Institute, University of Florida, Gainesville, Florida, United States of America; 2 Naval Medical Research Center-Asia/National Institute of Public Health, Phnom Penh, Cambodia; 3 National Institute of Public Health/Ministry of Health, Phnom Penh, Cambodia; Centers for Disease Control and Prevention, United States of America

## Abstract

In 2008, 800 adults living within rural Kampong Cham Province, Cambodia were enrolled in a prospective cohort study of zoonotic influenza transmission. After enrollment, participants were contacted weekly for 24 months to identify acute influenza-like illnesses (ILI). Follow-up sera were collected at 12 and 24 months. A transmission substudy was also conducted among the family contacts of cohort members reporting ILI who were influenza A positive. Samples were assessed using serological or molecular techniques looking for evidence of infection with human and avian influenza viruses. Over 24 months, 438 ILI investigations among 284 cohort members were conducted. One cohort member was hospitalized with a H5N1 highly pathogenic avian influenza (HPAI) virus infection and withdrew from the study. Ninety-seven ILI cases (22.1%) were identified as influenza A virus infections by real-time RT-PCR; none yielded evidence for AIV. During the 2 years of follow-up, 21 participants (3.0%) had detectable antibody titers (≥1∶10) against the studied AIVs: 1 against an avian-like A/Migratory duck/Hong Kong/MPS180/2003(H4N6), 3 against an avian-like A/Teal/Hong Kong/w312/97(H6N1), 9 (3 of which had detectible antibody titers at both 12- and 24-month follow-up) against an avian-like A/Hong Kong/1073/1999(H9N2), 6 (1 detected at both 12- and 24-month follow-up) against an avian-like A/Duck/Memphis/546/74(H11N9), and 2 against an avian-like A/Duck/Alberta/60/76(H12N5). With the exception of the one hospitalized cohort member with H5N1 infection, no other symptomatic avian influenza infections were detected among the cohort. Serological evidence for subclinical infections was sparse with only one subject showing a 4-fold rise in microneutralization titer over time against AvH12N5. In summary, despite conducting this closely monitored cohort study in a region enzootic for H5N1 HPAI, we were unable to detect subclinical avian influenza infections, suggesting either that these infections are rare or that our assays are insensitive at detecting them.

## Introduction

Since 2004, Cambodia has experienced more than 30 outbreaks of highly pathogenic avian influenza (HPAI) H5N1 virus among poultry and at least 47 human infections with 33 deaths [1], [2], [3]. By 2013, Cambodia had experienced more HPAI human infections and deaths than any other nation [4]. Live bird markets [5], movement of live poultry [5], humans bathing in ponds frequented by domestic ducks [6], and environmental exposures to H5N1 [7] have all been implicated as risk factors for these infections. Subsequently, Cambodia remains one of the regions of the world where HPAI H5N1 is enzootic among domestic poultry populations.

Despite the numerous documented outbreaks of H5N1 among poultry and the periodic human H5N1 cases that have been identified, previous seroepidemiology studies have estimated the seroprevalence of H5N1 antibodies to be relatively low (0%–2.6%) [6], [7], [8]. Each of these studies were conducted in areas where recent outbreaks of H5N1 in poultry had been molecularly confirmed, human cases identified, and the majority of participants reporting intense contact with poultry, all of which would intuitively suggest a greater risk for avian influenza transmission to humans. These studies, however, were limited as they only focused on H5N1 influenza virus and did not test for other avian influenza strains.

In 2008, we enrolled 800 rural villagers living in Kampong Cham Province, Cambodia, in a 2-year prospective epidemiological study for zoonotic influenza infections. In examining the cohorts' enrollment sera, we found evidence for subclinical infections with avian H9N2 infections [9]. This report documents our findings after 2 years of prospective study of this cohort.

## Materials and Methods

### Ethics statement

This study was approved by institutional review boards at the University of Iowa, University of Florida, Cambodia Ministry of Health National Ethics Committee, US Naval Medical Research Unit #2, Jakarta, Indonesia, and the US Naval Medical Research Center, Bethesda, MD. Each participant provided written informed consent.

### Study design

The study subjects, their locations, enrollment methods, questionnaires, and laboratory methods have been previously published [9]. Briefly, a total of 800 adults (≥20 years) living in 8 study sites, representing 9 rural Cambodian villages in Kampong Cham province, were enrolled in the study during 2008, and followed weekly for 24 months for evidence of influenza-like-illness (ILI). Sera and questionnaire data were collected at enrollment, 12 months, and 24 months.

### Weekly follow-up

During enrollment, cohort participants were given oral and written instructions, along with a digital thermometer, and were asked to inform study field staff, who conducted weekly home visits, upon developing signs and symptoms of an ILI. ILI was defined as acute onset of a respiratory illness with an oral (or equivalent from other body region) measured temperature ≥38°C, and a sore throat or cough for 4 or more hours.

### Investigating an influenza-like illness

When a possible ILI was reported to study staff, a home visit was performed within 72 hrs of notification. If the subject met the ILI case definition, a study nurse completed an ILI questionnaire and collected an acute serum sample and 2 respiratory swab specimens (nasal and pharyngeal). The swab specimens were stored in viral transport media and transported on ice packs at 4–8°C to the US Naval Medical Research Unit #2/National Institute of Public Health (NAMRU-2/NIPH) laboratory in Phnom Penh within 24 hrs after collection. Sixty days following the ILI investigation, study staff returned to the subject's home to collect a convalescent serum sample. If a participant developed a second case of ILI during the convalescent period, and the site principal investigator determined it was distinct from the original illness, the second ILI episode was considered a unique event and a new investigation was initiated.

### Family transmission study

A family investigation was initiated when an enrolled cohort study participant developed an acute influenza A infection, which was confirmed by real-time RT-PCR (rRT-PCR). During the home visit, family members were invited to sign an informed consent document and to participate in a family substudy of influenza transmission. A family member was defined as a person who lived in the same household of the study participant ≥20 days per month. Parents or guardians signed for family members <20 years of age. Family members who were between 7 and 20 years of age signed an assent form.

Study staff completed an ILI Case Household Form (one per household) through an interview with an adult household member. In addition, each consenting family member completed an ILI Case Contact Form to assess the individual's contact with the ill cohort member as well as recent animal exposures. Study staff collected an acute serum sample from each consenting subject. If a family member met the ILI case definition, nasal and pharyngeal swabs were also collected at the time of his/her enrollment. Family members were visited weekly for 9 weeks to monitor their possible development of ILI. If a family member met the ILI case definition, then respiratory swab specimens were collected. Sixty days following family members' enrollment, study staff returned to the home to collect a convalescent serum sample.

### Annual follow-up

Twelve and 24 months following enrollment, study staff visited participants' homes and completed an annual follow-up visit. Similar to enrollment procedures [9], participants provided a serum sample and completed a follow-up questionnaire that assessed changes to their demographics, health, or animal exposures during the past year. Exposures were defined as close contact within one meter of poultry or wild birds in the last 12 months. Serological analyses of the annual sera were performed to monitor changes in influenza antibody titers over time.

### Replacement enrollments

In order to maintain the number of active cohort subjects at around 800 participants, a replacement subject was enrolled after a cohort subject withdrew from the study for any reason. Study staff recruited the replacement subject from the non-enrolled household physically closest to the household from which the withdrawn subject came. Study staff randomly enrolled one adult from the replacement household following a similar process as the initial enrollment [9]. If all adults in that household refused to participate in the study, then study staff went to the next nearest household and continued in this way until an adult replacement cohort member was enrolled. After enrollment, weekly follow-up, as well as other activities (i.e. ILI investigation, family transmission study, etc.), began for that individual.

### Laboratory methods

Each respiratory specimen from prospective cohort subjects meeting the ILI case definition and from family member contacts were tested with rRT-PCR for influenza A at the NAMRU2/NIPH laboratory, within 72 hours of collection. Sera and ILI respiratory swab aliquots were preserved at −80°C and transported on dry ice to the University of Florida for testing. ILI swabs were again screened with rRT-PCR for influenza A. Sera were tested for evidence of human, swine, and avian influenza infections over time (Table 1). Influenza viruses, viral antigens, and control antisera were obtained from acknowledged collaborators, Biodefense and Emerging Infections (BEI) Research Resources Repository, or through the Influenza Reagent Resource (IRR) program of the US CDC.

**Table 1:** Viruses used in serological studies.

Avian viruses	Swine viruses
A/Migratory duck/Hong Kong/MPS180/2003(H4N6)	A/Swine/Lutol/3/2000(H1N1)*
A/Nopi/Minnesota/07/462960-2(H5N2)	A/Swine/Gent/7625/1999(H1N2)*
A/Teal/Hong Kong/w312/1997(H6N1)	A/Swine/Flanders/1/1998(H3N2)*
A/Env/Hong Kong/MPB127/2005(H7N7)	**Human viruses**
A/Migratory duck/Hong Kong/MP2553/2004(H8N4)	A/Brisbane/59/2007(H1N1)*
A/Migratory duck/Hong Kong/MPD268/2007(H10N4)	A/Mexico/4108/2009(pandemic H1N1)*
A/Chicken/New Jersey/15906-9/1996(H11N1)	A/Brisbane/10/2007(H3N2)*
A/Duck/Alberta/60/1976(H12N5)	A/Cambodia/R0404050/2007(H5N1)^†‡^
	A/Hong Kong/1073/99(H9N2)^†^

Unless otherwise indicated serologic study was performed using the microneutralization technique.

Viruses were selected from among our virus library considering their H type, and their year and geographical area of collection for the closest match with viruses likely to be circulating in Cambodia. Unfortunately, we did not have a large collection of viruses from Cambodia from which we might choose. *Virus studied with hemagglutination inhibition assay; ^†^Virus of avian origin; ^‡^Highly pathogenic virus.

#### Real-time RT-PCR influenza assay

Viral RNA was isolated from 140 µl of each swab specimen and processed using the Qiagen: QIAamp Viral RNA Mini Kit (Qiagen Inc., Valencia, California) following a mini-spin protocol. Contaminants were washed away by two wash buffers and the RNA eluted in 50 µl of elution buffer. Specimens were screened for the presence of influenza A viral RNA using the Centers for Disease Control and Prevention's (CDC's) Human Influenza Virus Real-Time RT-PCR Detection and Characterization Panel [10]. The primer and dual labeled hydrolysis probes in this system are capable of universal detection of influenza A virus while subtyping primer and probe sets are designed to specifically detect contemporary human A/H1, human 2009 pandemic H1N1, human A/H3, and avian A/H5 (Asian lineage) influenza viruses. Each extraction run included a mock extraction control to provide a secondary negative control to validate the extraction procedure and reagent integrity. The human RNase P gene primer set was used as an internal positive control for human RNA in each sample. Specimens that were rRT-PCR positive for generic influenza type A were further evaluated with a rRT-PCR procedure specific for human H1, H3, and H5, as well as 2009 pandemic H1. Swab samples positive for influenza A, but unable to be subtyped, were cultured in Madin-Darby canine kidney (MDCK) cells and passaged twice in an attempt to amplify the virus for further study.

#### Hemagglutination inhibition (HI) assay

We employed the WHO-recommended HI assay [11] to test for serum antibodies against human and swine influenza A viruses. Influenza virus strains were grown in MDCK cells or fertilized eggs. Sera were pre-treated with receptor destroying enzyme and hemabsorbed with either guinea pig or turkey erythrocytes. For seasonal human influenza virus strains, guinea pig erythrocytes were used in assay plates with round bottom or “U” shaped wells. For swine influenza viruses, turkey erythrocytes were used in plates with conical bottom or “V” shaped wells. Titer results were reported as the reciprocal of the highest dilution of serum that inhibited virus-induced hemagglutination of a 0.65% (guinea pig) or 0.50% (turkey) solution of erythrocytes [12].

#### Microneutralization (MN) assay

A WHO-recommended MN assay adapted from that reported by Rowe [13], [14], [15] was used to detect human antibodies against avian viruses. The viruses were grown in fertilized eggs. Sera were first screened at a dilution of 1∶10. Positive specimens were then titered out in duplicate by examining 2-fold serial dilutions from 1∶10 to 1∶1280 in virus diluent [85.8% minimum essential medium (Invitrogen, Carlsbad, CA), 0.56% BSA, 25 mM HEPES buffer (Invitrogen), 100 mg/l streptomycin (Invitrogen), and 100,000 units/l penicillin (Invitrogen)]. Virus neutralization was then performed by adding 100 TCID_50_ of virus to the sera. The Reed Muench method was used to determine the TCID_50_/100 µL [16]. MDCK cells in log phase growth were adjusted to 2.0×10^5^ cells/mL with virus diluent. One hundred microliters of this suspension of cells was added to each well, after which plates were incubated at 37°C with 5% CO_2_ for 24 hours. Plates were washed twice with PBS, fixed with cold 80% acetone, and incubated at room temperature for 10 minutes. Influenza on the fixed monolayers was then quantified by influenza A nucleoprotein-specific indirect ELISA. The plates were washed with phosphate buffered saline containing 0.05% Tween 20, between each antibody addition, after one hour incubation at room temperature. Following the final wash, 0.1 ml of 3,3',5,5'- tetramethylbenzidine (TMB) (KPL 50-76-03)(Kirkegaard & Perry Laboratories Inc., Gaithersburg, Maryland) was added and incubated at room temperature for 10 min. The peroxidase reaction was stopped by the addition of 0.1 ml of 1N sulfuric acid. The optical density of the plates was read at 450 nm. The ELISA endpoint titer was expressed as the reciprocal of the highest dilution of serum with optical density (OD) less than X, where X  =  [(average OD of virus control wells) + (average OD of cell control wells)]/2. Test cells with an OD>2 times the cell control OD mean were considered positive for virus growth. A back titration of the virus antigen was run in duplicate and only accepted when both replicates had matching results.

### Statistical methods

Study outcomes were evidence of previous or acute influenza A virus infections. Acute influenza infection was defined as either a) isolation of influenza virus from a respiratory specimen obtained when a patient had an ILI, b) rRT-PCR evidence of influenza from such specimens, or c) a fourfold or greater rise in antibody titer against influenza viruses for paired ILI or annual follow-up sera with a threshold antibody titer of ≥1∶80 for avian viruses. As done previously [15], [17], [18], [19], [20], a HI titer ≥1∶40 was accepted as evidence of human or swine influenza virus infection or human influenza vaccination.

## Results

Between April and October 2008, field staff enrolled a total of 800 participants (100 from each of 8 sites). Participant demographics at enrollment have previously been reported [9]. Over the 24-month follow-up period, 89 originally enrolled participants withdrew from the study. Overall, 77 replacement enrollments were added, of which 4 also withdrew after the 12-month follow-up period. A total of 800 participants (100%) completed the 12-month annual follow-up and 784 participants (98%) completed the 24-month annual follow-up visit. Overall, 708 participants (89%) remained enrolled for the entire study duration by completing enrollment and both 12- and 24-month follow-up visits. A total of 438 ILI investigations were conducted among 284 cohort subjects (97 subjects experienced >1 unique ILI event). In addition, 364 household contacts were enrolled in the family transmission study; 89 ILI episodes were reported among 85 (23.4%) contacts (4 contacts experienced multiple ILIs).

### Acute human influenza A infections

**Figure 1:**
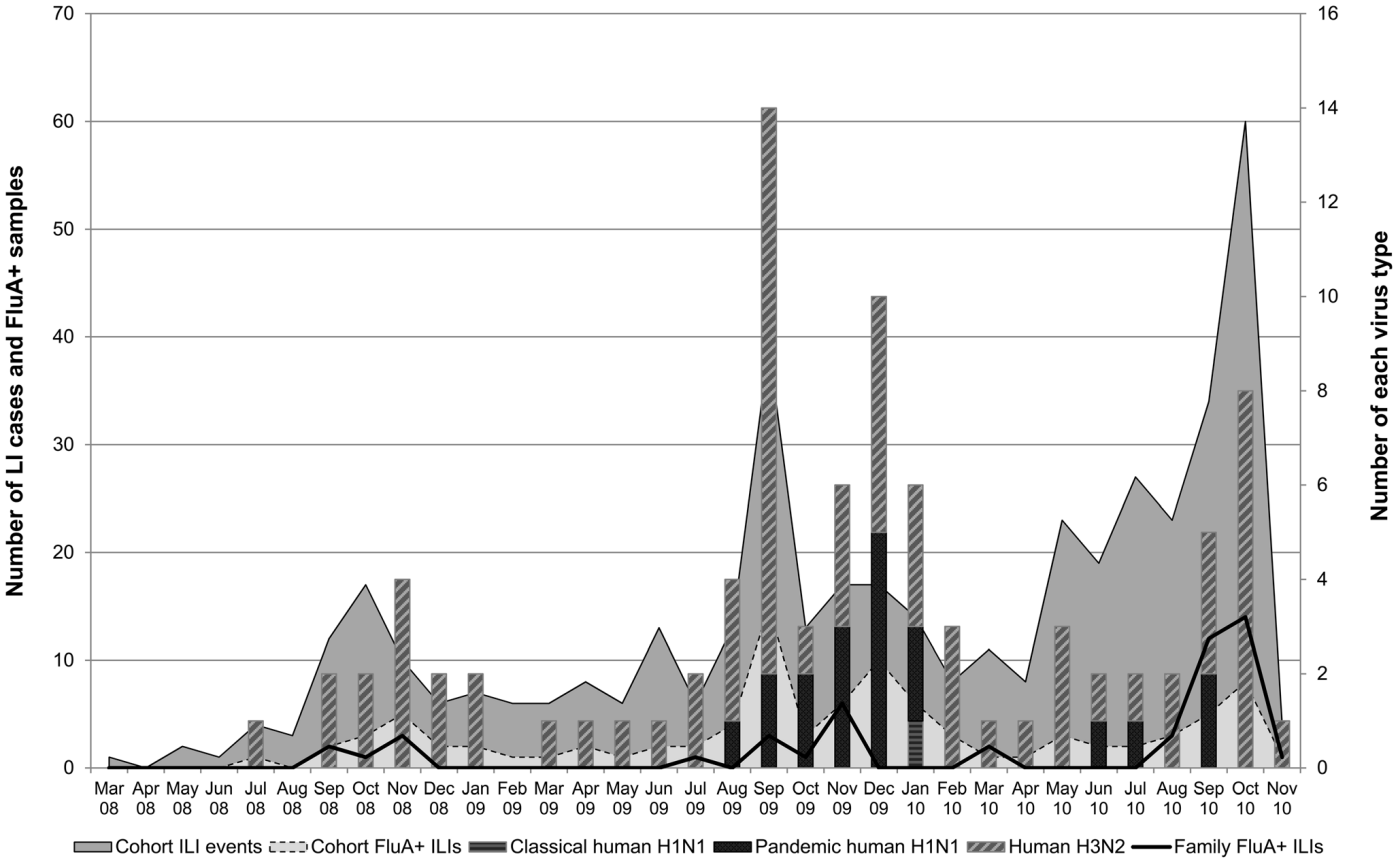
Reported influenza-like illnesses with real-time RT-PCR results of respiratory swabs collected from study cohort members and their family contacts at time of illness; June 2008 - November 2010; Kampong Cham, Cambodia.

rRT-PCR analyses were performed on nasal and pharyngeal swabs collected during ILI episodes (Figure 1). Among the cohort subjects, 97 (22.1%) of the swabs taken from the 438 reported ILI events were positive by rRT-PCR for influenza A virus, corresponding to an incidence rate of 123 per 1000 among individuals who completed follow-up. Of the 89 reported ILIs among family contacts, 49 (55.1%) were rRT-PCR positive for influenza A, corresponding to an incidence rate of 135 per 1000. Among the 146 total participants positive for influenza A, 102 subjects were positive for human H3N2 influenza A virus, 40 had influenza A(H1N1)pdm09 virus, and 1 had classical human H1N1 influenza A virus.

Fifty-nine (60.8%) of the 97 rRT-PCR positive cohort ILI events had a corresponding 4-fold rise in HI titer. Of the remaining 341 ILI events among the cohort, negative by rRT-PCR, 31 (9.1%) had serological evidence of influenza infection. As paired sera were collected from all household contacts who volunteered, regardless of experiencing ILI symptoms, 9 (10.6%) of the 85 symptomatic family members and 26 (9.3%) of the 279 asymptomatic family members experienced a 4-fold or greater increase in HI titers from the acute to convalescent blood draws. When examining serological reactivity of the 146 influenza positive rRT-PCR results by subtype, 54 (52.9%) of the 102 subjects positive for H3N2 had a 4-fold or greater HI titer increase, 10 (25.0%) of the 40 subjects rRT-PCR positive for pH1N1 had a 4-fold or greater increase in HI titer, and the 1 subject rRT-PCR positive for classical H1N1 did not have a corresponding increase in HI titer.

Numerous subclinical or mild influenza A virus infections, not detected as ILIs, were identified through 4-fold or greater increases in HI titers amongst the cohort by analyses of annual follow-up sera. There were 103 subjects showing evidence of infection with classical human H1N1, of which 64 (62.1%) did not report an ILI during the respective follow-up period. A total of 326 subjects' HI titers increased ≥4-fold against human H3N2 influenza, of which 231 (70.9%) did not report an ILI during the respective follow-up time. Against the pandemic H1N1 influenza virus, annual HI titers increased ≥4-fold between annual bleeds among 53 subjects, of which 33 (62.3%) did not report an ILI.

### Avian influenza virus infections

One HPAI H5N1 virus infection was detected by molecular analyses of the respiratory swabs collected during an ILI investigation; this subject's paired ILI sera had no evidence of elevated neutralizing antibodies against the H5N1 virus. Among all cohort subjects sampled at 0, 12 and 24 months, there was no serological reactivity against the H5N1 virus. There was, however, limited evidence of serological reactivity against low-pathogenic avian influenza (LPAI) viruses, including low MN titer activity against H4N6, H6N1, H9N2, H11N1, and H12N5 (Table 2). At enrollment, of the subjects that had neutralizing antibodies ≥1∶80 against LPAI viruses, one was AvH9N2 and the other AvH12N5. In addition, of the subjects with elevated titers at the annual follow-up encounters, one subject had a ≥4-fold increase in neutralizing antibody titer against AvH12N5, with a minimum threshold titer of 1∶80 (Table 3).

**Table 2:** Number of study subjects with elevated microneutralization titers against low pathogenic avian influenza viruses, Kampong Cham Province, Cambodia, 2009–10.

Antibody titer	Avian Influenza Virus														
	H4N6			H6N1			H9N2			H11N1			H12N5		
	0 mo	12 mo	24 mo	0 mo	12 mo	24 mo	0 mo	12 mo	24 mo	0 mo	12 mo	24 mo	0 mo	12 mo	24 mo
1∶10	-	-	1	-	1	2	2	3	3	1	2	2	-	-	-
1∶20	-	-	-	-	-	-	6	3	4	-	2	2	-	-	-
1∶40	-	-	-	-	-	-	-	-	-	-	1	-	-	-	1
1∶80	-	-	-	-	-	-	-	1	-	-	-	-	-	1	-
1∶160	-	-	-	-	-	-	1	-	-	-	-	-	1	-	-
Total:	0	0	1	0	1	2	9	7	7	1	5	4	1	1	1

H4N6 = A/Migratory duck/Hong Kong/MPS180/2003(H4N6); H6N1 = A/Teal/Hong Kong/w312/97(H6N1); H9N2 = A/Hong Kong/1073/99(H9N2); H11N1 = A/Chicken/New Jersey/15906-9/1996(H11N1); H12N5 = A/Duck/Alberta/60/1976(H12N5).

**Table 3:** Study participants with detectable microneutralization assays titers against avian influenza viruses at enrollment, 12- and 24-month follow-up, Kampong Cham Province, Cambodia, 2008–10.

Virus/ID	0 Months	12 Months	24 Months
H4N6			
0276	<1∶10	<1∶10	1∶10
H6N1			
0021	<1∶10	<1∶10	1∶10
0437	<1∶10	1∶10	<1∶10
0719	<1∶10	<1∶10	1∶10
H9N2			
0035	1∶160	1∶80	1∶20
0138	1∶20	<1∶10	<1∶10
0316	<1∶10	<1∶10	1∶20
0355	<1∶10	1∶10	<1∶10
0392	1∶10	1∶10	<1∶10
0582	<1∶10	1∶20	1∶20
0661	1∶10	1∶10	<1∶10
0707	1∶20	1∶20	1∶20
0734	1∶20	<1∶10	1∶10
0736	1∶20	<1∶10	<1∶10
0748	1∶20	1∶20	<1∶10
0768	1∶20	<1∶10	Missing*
H11N1			
0045	<1∶10	<1∶10	<1∶10
0171	1∶10	1∶10	<1∶10
0301	<1∶10	<1∶10	1∶20
0306	<1∶10	1∶20	<1∶10
0592	<1∶10	1∶40	1∶20
0666	<1∶10	1∶20	<1∶10
0711	<1∶10	1∶10	<1∶10
H12N5			
0311	1∶160	<1∶10	1∶40
0312	<1∶10	1∶80	<1∶10

*Lost to follow-up.

### Swine influenza virus infections

While some positives were identified in tested samples, it appeared that the SIV HI assay results were heavily confounded by cross-reacting antibodies from human influenza viruses, making it difficult to interpret results.

## Discussion

While new influenza infections were frequent among the cohort and cohort family members, we did not serologically detect AIV infections among 800 rural Cambodian villagers and 364 close family contacts suggesting that either AIV infections are rare or our methods are insensitive. With the exception of the single H5N1 infection, there were no other AIV infections detected from clinically ill participants monitored weekly for ILI. Serological evaluation of cohort members to detect subclinical or mild AIV infection revealed only two subjects to have neutralizing antibodies ≥1∶80 against LPAI viruses at enrollment, and only one individual to have a 4-fold increase in antibody titer against AvH12N5 during follow-up, with a 12-month microneutralization antibody titer of 1∶80. This individual reported poultry exposure and did not experience an ILI, which could suggest a possible sub-clinical infection, though cross-reactivity cannot be entirely ruled out.

There was considerable evidence of human influenza infection among the study population. Human influenza A detection among ILI cases showed clear seasonality with a peak between August and November in each study year. It is thought that influenza seasonality in Cambodia is closely related to the rainy season, which begins around June and peaks around October [21]. This seasonality is consistent with previously published reports [21], [22]. In addition, human influenza A (H3N2) appeared to be the more prevalent virus subtype detected among ILI samples. While subclinical pandemic H1N1 infections have been widely recognized [23], [24], the high prevalence of subclinical H1N1 (62.1%) and H3N2 (70.9%) infections, as evidenced by a 4-fold rise in HI titer, was a bit unexpected. Previous longitudinal studies have estimated asymptomatic infections of pandemic H1N1, seasonal H1N1,and H3N2 influenza infections ranging from between 25%–36% [25], [26], [27]. While it is possible that the high proportion of asymptomatic seroconversions identified in this study might be at least partially explained by cross-reacting heterotypic antibodies [28], it seems more likely that these rural villagers were more tolerant of mild symptoms of influenza infections and thus less likely to report same as compared to the participants in other studies.

Since 2004, a number of AIV infections have been detected among poultry and humans in Cambodia, particularly HPAI H5N1, with 5 poultry outbreaks occurring in 2011 after the final follow-up of this cohort [1], [2], [3]. Thus, as in other highly endemic areas such as Indonesia and Vietnam, human exposures with HPAI remain enigmatic. It seems possible that our serological assays lack sensitivity. It is also possible that our cohort members were not exposed to high risk environments such as the live bird markets in densely populated areas, which have been identified as a common source of AIV transmission throughout Cambodia [29], [30]. To better understand AIV transmission among poultry, it would have been advantageous to also test participants' flocks for evidence of AIV infection. We might have also increased the sera sampling from once a year to once every six months, since it has been previously reported that antibody titers acquired through subclinical AIV infections may wane within one year [31], suggesting such shorter test intervals may be necessary to capture subtle changes in antibody response. Additionally, a WHO-recommended conservative cut-off of 1∶80 was used, which could have caused us to miss some true positive cases.

As the study period was between 2008 and 2010, we were able to capture epidemiological data regarding the introduction of the 2009 pandemic influenza strain (pH1N1) into Cambodia. Specifically, the HI assay revealed no antibody response to pH1N1 among cohort members upon enrollment (2008), however, at 12- and 24-month follow-ups 6 (0.8%) and 49 (6.3%) of cohort members had antibody titers ≥1∶40, representing a marked increase over the 2-year study period. Study results also indicated that pH1N1 became a more prevalent virus subtype in 2010, an epidemiological characteristic consistent with other reports [32].

This study had a number of limitations. As previously discussed, antibodies acquired through subclinical AIV infections likely wane within one year [31], for which testing of only 12- and 24-month annual sera may have missed. The study was further limited in that we did not study children, a population often at increased risk for AIV infection. For instance, a report by Humphries-Waa et al. revealed that 61% of the patients with HPAI H5N1 identified during 2005–2011 were under the age of 18 [2]. While children were included in the family contact enrollments, their infections could have been missed as it was common for illnesses in children to have preceded that of cohort members. Recall bias could have affected the reliability of our questionnaire data, as it is likely difficult for participants to accurately remember their prior exposure to poultry and other animals, as well as questions related to their medical history. Lastly, each of the influenza strains used in the laboratory analysis may not have been representative of the strains circulating in Cambodia during the study period.

This report describes one of few large prospective studies of AIV infection in rural Asia. Overall, the study was very resource-intensive and required continual close monitoring to ensure proper execution. Even though surveillance was able to capture useful data related to seasonal influenza patterns, it seems likely that larger cohort studies involving greater geographic areas and more frequent sampling are likely necessary to better understand subclinical AIV transmission dynamics - an effort that just may not be feasible given the resources required.
